# Evaluation of Crystallographic Data with the Program DIAMOND

**DOI:** 10.6028/jres.101.023

**Published:** 1996

**Authors:** Günter Bergerhoff, Michael Berndt, Klaus Brandenburg

**Affiliations:** Institute for Inorganic Chemistry, University of Bonn, D - 53121 Bonn, Germany

**Keywords:** crystal structure, database, molecular graphics

## Abstract

The new crystal structure information system DIAMOND is presented. It handles all kinds of crystallographic databases on PCs including SHELX files and Chemical Information Files (CIF’s). DIAMOND, because of its graphics capability, is a powerful tool for establishing structural relationships and for evaluating crystallographic data.

## 1. Introduction

Databases are now well established but there are still the old demands. Databases should be complete, up-to-date, correct, user-friendly, and versatile. Experience shows that this goal can be reached asymptotically only, and that the acceptance of databases by the scientific community runs parallel to the curve which describes the development and improvement of the databases. This means the financial background for maintaining databases also depends on the power of the databases.

Databases consist of two elements: i) the actual data ii) programs to handle the data. We have several well-established data centers responsible for collecting data in their specific fields. I hope they try to be complete, if not then users must press them to fill the gaps.

To be up-to-date new methods of electronic transfer to fill the data pools are used more and more. Journals and authors should learn to take advantage of these facilities.

Correctness is a much more complex problem. Each database producer has its own checking routines, nevertheless errors are unavoidable. There are two types: i) first those made by the database producer, eg., small misprints or misinterpretation of inadequately described structures, especially in the older literature, and ii) second errors published by the author and—perhaps—recognized in more recent papers. I think you all know the important work of Richard Marsh in finding correct space groups for older structure determinations. Silicon dioxide is another example. The ICSD contains 133 determinations between 1925 and 1992 and for tridymite there are still eight different descriptions. Without appropriate scientific background database producers cannot overcome the existing problems and in any case, users must help to detect errors.

For the ICSD, the responsibility for completeness, accuracy and correctness is now at Fachinformations-zentrum Karlsruhe and Gmelin-Institute. We at Bonn University try to contribute to user-friendliness and versatility.

## 2. General Retrieval Features

In the beginning of the database era programs to handle databases were very complicated and experts and big machines were needed. There are still such databases, but today information science provides tools which allow us to follow the way of thinking of the chemist and the crystallographer on anyone’s PC with WINDOWS. No longer is it necessary to know cryptic commands to look for the desired information and no longer is it necessary to learn different commands for different databases. The database system DIAMOND in principle handles all existing structural databases including your own private data pool with the same self-indexing and menu-driven retrieval system designed for the special purpose of the structural chemist. A maximum of user-friendliness would be achieved if all databases were reformatted to the common DIAMOND format. It is not a technical problem. At present we offer the possibility to collect downloaded files of your special interest from the different databases in order to make use of DIAMOND. The collection can include SHELX-files or CIFs from your own production.

The retrieval is then possible for all features of structural databases which are explicitly given in the internal data by simple clicks on the appropriate field:

- chemical elements, their combinations, and oxidation states (in the ICSD only),
- symmetry properties,
- bibliographic data, etc.

Database programs must be able to give more information than what is present explicitly in the stored data. A versatile database program has to support all ideas of the user. First of all it has to translate formal data like unit cell dimensions, symmetry elements and atomic coordinates to a picture of the retrieved structure. With DIAMOND you can create all forms of pictures you want by a click with the mouse.

**Fig. 1:**
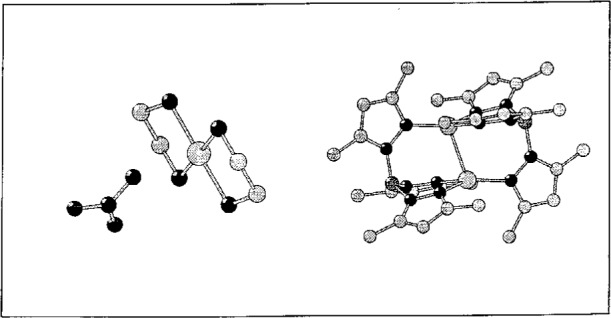
Molecules from CSD: Copper(ii) bis(ethylenediamine) nitrate (Ref. code COPDEN, left) and bis(μ_2_-Hydrido-tris(3,5-dimethyl-1-pyrazolyl)borato)-dicopper (BMPZCU).

Molecules, ie. distinct entities described by bonds, are developed from the asymmetric set of coordinates automatically (see Fig. 1).

**Fig. 2:**
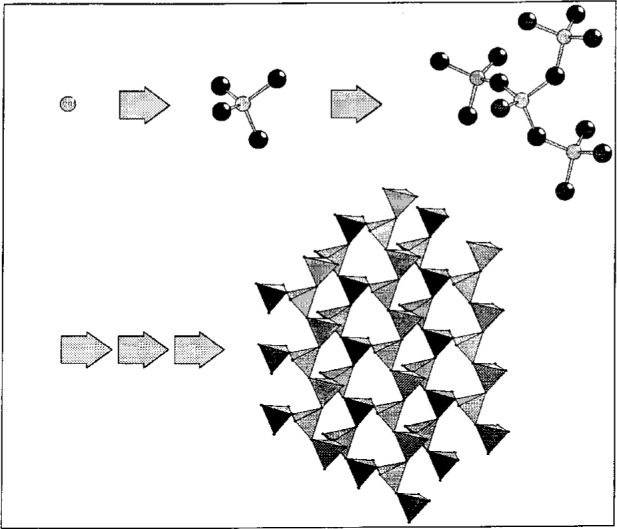
Building up infinite layers.

Infinite or polymeric structures, typical for inorganics and alloys, are built up from a selected starting atom. For better understanding atomic groups can be represented by polyhedra (see Fig. 2).

**Fig. 3:**
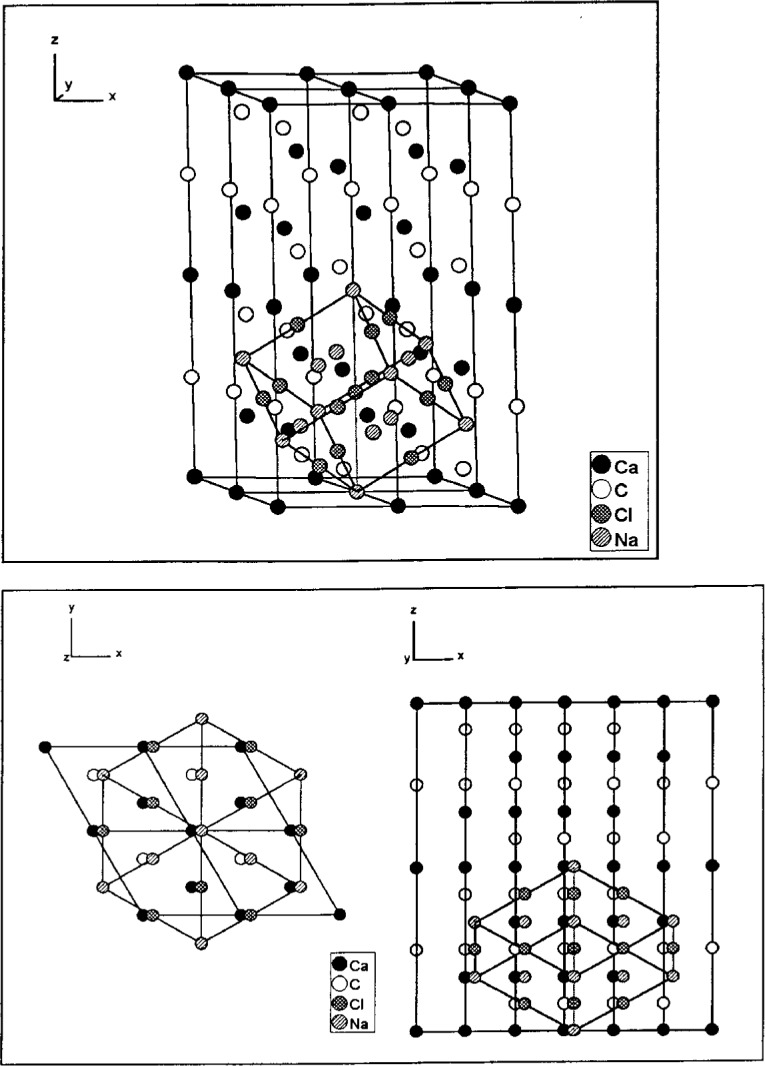
Superimposing CaCO_3_ and distorted NaCl.

For visual comparison different structures can be put into windows side-by-side or can be superimposed by distortion and enlargement. The pair CaCO_3_ - NaCl is a well known example (see Fig. 3).

**Fig. 4:**
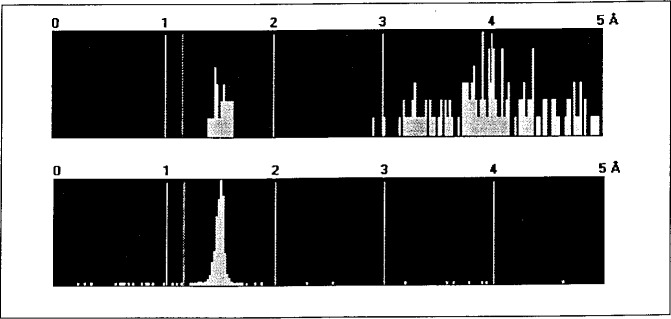
Histograms of distances in Sr_2_P_6_O_17_ and all P–O distances.

Of course these features are possible only by selecting atomic distances as predefined bonds. This is fairly easy in organic molecules, but it is difficult in many inorganic structures and alloys. Therefore before you start drawing you examine the histogram of the distances in the structure in question and set the appropriate limits. If you are not sure about the general range of distances you look at the general histogram collected from all entries in the ICSD (see Fig. 4).

For special purposes you can measure distances by a click and draw what you want or delete what is not valid. This are only some of the features for creating pictures as you like or forming search strategies for items which are in your data implicitly.

## 3. Structural Relationships

In inorganic chemistry, including alloys related structures are very common. Besides the formal analogy of AB-, ABX3-structures, etc., e.g.:
CaF_2_⇒AX2CaTiO_3_⇒ABX3Ca_2_SiO_4_⇒AB2X4K_4_[Fe(CN)_6_]⇒AB4C6X6Ag_2_S⇒A2XTi_2_N⇒NO2Ca_5_(PO_4_)_3_(OH)⇒A3B5X13Be_3_Al_2_(Si_6_O_18_)⇒A2B3C6X18isotypism is one of the relationships often described in textbooks with examples. But there is no definition which is so well done that it can be translated to a program. The first step has been done by Parthé and Gelato [1] by standardization of crystal structure descriptions and defining isopointal structures. When applied in general to the ICSD it turned out that it is not a general solution. There are to many cases when it does not work. We have to compare each structure with each other within an isopointal group and to calculate the mean difference, *Δ*, (*M* = multiplicity) between the most similar set of coordinates of two structures.

$$\Delta =\frac{\Sigma \left[M\cdot \sqrt{\left(\delta x^{2}+\delta y^{2}+\delta z^{2}\right)}\right]}{\Sigma M}$$

In such a way we get *Δ*-values for the 9 existing members of the isopointal group 19/a3, as shown in Table 1.

**Fig. 5:**
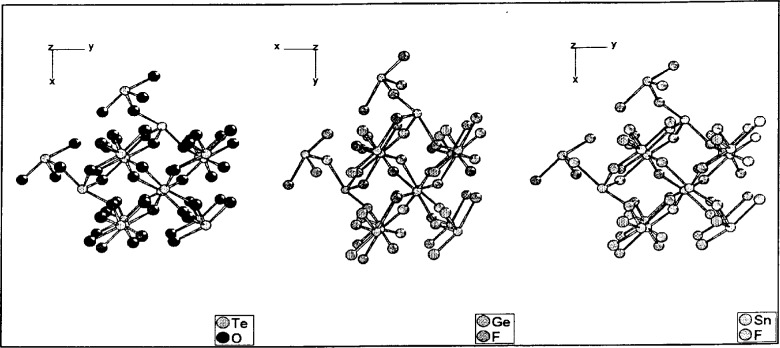
Comparison of GeF_2_–TeO_2_–SnF_2_.

Low *Δ*-values show a stronger relationship. For the triple TeO_2_–GeF_2_–SnF_2_ (*Δ*-values printed in bold face), DIAMOND can give more insight into the relationship by selecting four bonds from metals to non-metals from the specific histogram, similar patterns are built for all three structures (see Fig. 5).

The differences are shown in Table 2 by a detailed calculation of distances (Å) and angles (°).

It is only a question of time (and money) before we have reorganized the ICSD to a SICS (Standardized Inorganic Crystal Structures) running in parallel to ICSD. This will not only allow us to make isotypic structures searchable setting a certain degree *Δ* of isotypism, but also to clean the ICSD for errors which can be detected by comparison.

We know that there are many more relationships between inorganic structures, e.g., subgroup-supergroup relationships. Using the program TRANSFORM an aristotype can be transformed to a lower symmetrical space group forming another isopointal group. Then it can be compared with the other members of this new group. Once more the *Δ*-values will describe the similarity of the structures in question. It has been my concern to show you the necessity and the possibilities to improve databases in order to get not only a maximum of acceptance by the scientific community but also a tool which will give a much more thorough understanding of the solid state. For a general solution of this problem a database version of the International Tables for Crystallography would be very welcome. We maintain information about our activities on the WWW: http://www.rhrz.uni-bonn.de/~unc442/diamond.html.

## Figures and Tables

**Table 1:** *Δ*-values for the isopointal group 19/a3

	CdF(OH)	Sn F_2_	Ag_2_ Se	Ge F_2_	Zn (O H)_2_	Be (O H)_2_	Hg Br Cl	Te O_2_	Hg F (O H)
Cd F (O H)		0.1017	0.1138	0.1014	0.1420	0.1012	0.1616	0.0840	0.0536
Sn F_2_	0.1017		0.1208	**0.0755**	0.1083	0.0811	0.1924	**0.0518**	0.1053
Ag_2_ Se	0.1138	0.1208		0.1302	0.1505	0.1399	0.1976	0.1330	0.0978
Ge F_2_	0.1014	**0.0755**	0.1302		0.1128	0.0874	0.1715	**0.0459**	0.1103
Zn (O H)_2_	0.1420	0.1083	0.1505	0.1128		0.0814	0.1341	0.1073	0.1188
Be (O H)_2_	0.1012	0.0811	0.1399	0.0874	0.0814		0.1586	0.0820	0.0960
Hg Br Cl	0.1616	0.1924	0.1976	0.1715	0.1341	0.1586		0.1685	0.1470
Te O_2_	0.0840	**0.0518**	0.1330	**0.0459**	0.1073	0.0820	0.1685		0.0867
Hg F (O H)	0.0536	0.1053	0.0978	0.1103	0.1188	0.0960	0.1470	0.0867	

**Table 2:** Bond distances and angles

‘Ge	1′	91.67 °	‘F	1′	1.789 Å	‘F	2′	1.906 Å
		85.55 °	‘F	1′	1.789 Å	‘F	2′	2.091 Å
		82.41 °	‘F	1′	1.789 Å	‘F	1′	2.563 Å
		84.81 °	‘F	2′	1.906 Å	‘F	2′	2.091 Å
		83.33 °	‘F	2′	1.906 Å	‘F	1′	2.563 Å
		162.82 °	‘F	2′	2.091 Å	‘F	1′	2.563 Å
‘F	1′	136.74 °	‘Ge	1′	1.789 Å	‘Ge	1′	2.563 Å
‘F	2′	157.38 °	‘Ge	1′	1.906 Å	‘Ge	1′	2.091 Å
‘Te	1′	80.28 °	‘O	2′	1.912 Å	‘O	1′	2.034 Å
		102.30 °	‘O	2′	1.912 Å	‘O	1′	2.044 Å
		87.20 °	‘O	2′	1.912 Å	‘O	2′	2.110 Å
		89.27 °	‘O	1′	2.034 Å	‘O	1′	2.044 Å
		162.90 °	‘O	1′	2.034 Å	‘O	2′	2.110 Å
		82.03 °	‘O	1′	2.044 Å	‘O	2′	2.110 Å
‘O	1′	135.48 °	‘Te	1′	2.034 Å	‘Te	1′	2.044 Å
‘O	2′	134.39 °	‘Te	1′	1.912 Å	‘Te	1′	2.110 Å
‘Sn	1′	78.85 °	‘F	1′	1.893 Å	‘F	1′	2.257 Å
		73.75 °	‘F	1′	1.893 Å	‘F	2′	2.398 Å
		104.01 °	‘F	1′	1.893 Å	‘F	2′	2.406 Å
		147.07 °	‘F	1′	2.257 Å	‘F	2′	2.398 Å
		105.24 °	‘F	1′	2.257 Å	‘F	2′	2.406 Å
		98.89 °	‘F	2′	2.398 Å	‘F	2′	2.406 Å
‘F	1′	156.65 °	‘Sn	1′	1.893 Å	‘Sn	1′	2.257 Å
‘F	2′	111.81 °	‘Sn	1′	2.398 Å	‘Sn	1′	2.406 Å
